# Filtering out parasites: sand crabs (*Lepidopa benedicti*) are infected by more parasites than sympatric mole crabs (*Emerita benedicti*)

**DOI:** 10.7717/peerj.3852

**Published:** 2017-09-22

**Authors:** Zen Faulkes

**Affiliations:** Department of Biology, The University of Texas Rio Grande Valley, Edinburg, TX, United States of America

**Keywords:** Crustacean, Parasite manipulation of behavior, Nematode, Parasite, Cestode, Hippoidea, Digging

## Abstract

Two digging decapod crustaceans, the sand crab species *Lepidopa benedicti* and the mole crab species *Emerita benedicti*, both live in the swash zone of fine sand beaches. They were examined for two parasites that infect decapod crustaceans in the region, an unidentified nematode previously shown to infect *L. benedicti*, and cestode tapeworm larvae, *Polypocephalus* sp., previously shown to infect shrimp (*Litopenaeus setiferus*). *Lepidopa benedicti* were almost always infected with both parasite species, while *E. benedicti* were rarely infected with either parasite species. This difference in infection pattern suggests that tapeworms are ingested during sediment feeding in *L. benedicti*, which *E. benedicti* avoid by filter feeding. Larger *L. benedicti* had more *Polypocephalus* sp. larvae. The thoracic ganglia, which make up the largest proportion of neural tissue, contained the largest numbers of *Polypocephalus* sp. larvae. Intensity of *Polypocephalus* sp. infection was not correlated with how long *L. benedicti* remained above sand in behavioural tests, suggesting that *Polypocephalus* sp. do not manipulate the sand crabs in a way that facilitates trophic transmission of the parasite. *Litopenaeus setiferus* may be a primary host for *Polypocephalus* sp., and *L. benedict* may be a secondary, auxiliary host.

## Introduction

Parasites can be generalists that infect many host species, or specialists that infect only a small number of host species, or even just one host species. (Poulin, 2007; Schmid-Hempel, 2011; Loker & Hofkin, 2015). A benefit of being a specialist may be increased adaptation to a host species. Specialization should be favoured in endoparasites that manipulate host behaviour (Adamson & Caira, 2011; Fredensborg, 2014), because the nervous systems generating behaviour are probably anatomically and physiologically more variable than other types of tissue (Bullock, 1993; Bullock, 2004; Bullock, 2006). Behavioural manipulation often manifests as parasite induced tropic transmission (PITT), in which parasites with multiple host life cycles change the behaviour of one host in such a way as to enhance the likelihood of the host being eaten by a predatory species that is the next host in the parasite’s life cycle (Moore, 2002; Lafferty & Shaw, 2013).

**Figure 1:**
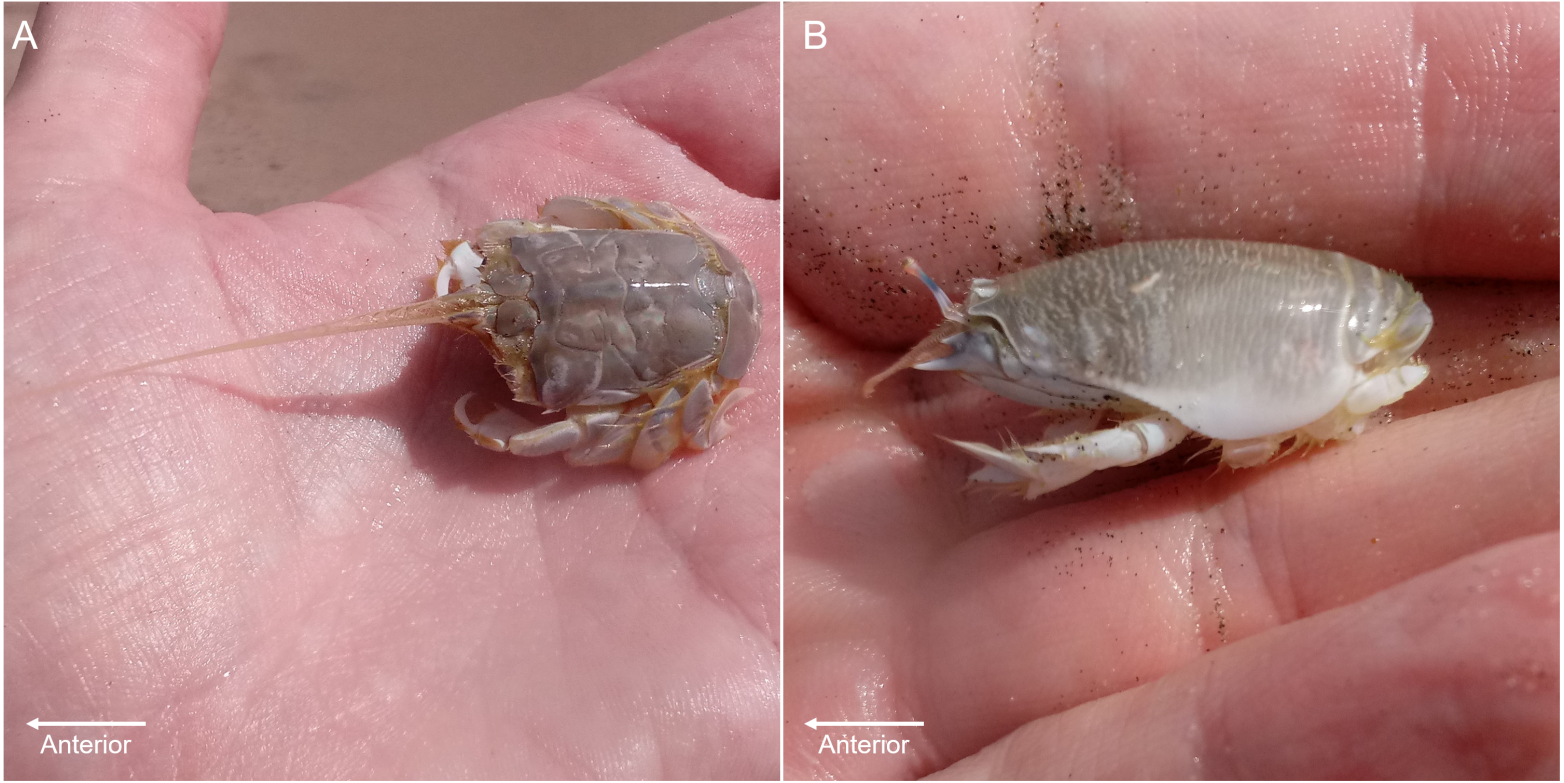
Digging crab species. (A) *Lepidopa benedicti*. (B) *Emerita benedicti*.

Sand crabs (*Lepidopa benedicti*) and mole crabs (*Emerita benedicti*) are digging anomuran crustaceans in the same superfamily (Hippoidea) (Fig. 1), which are both found in the swash zones of sandy beaches in the Gulf of Mexico. Given that they are relatively closely related and found in the same habitat, it is a reasonable hypothesis that they might have similar parasites to each other. *Lepidopa benedicti* is often infected by an unidentified nematode species that does not appear to manipulate host behaviour (Joseph & Faulkes, 2014), which might also infect *E. benedicti*.

**Figure 2:**
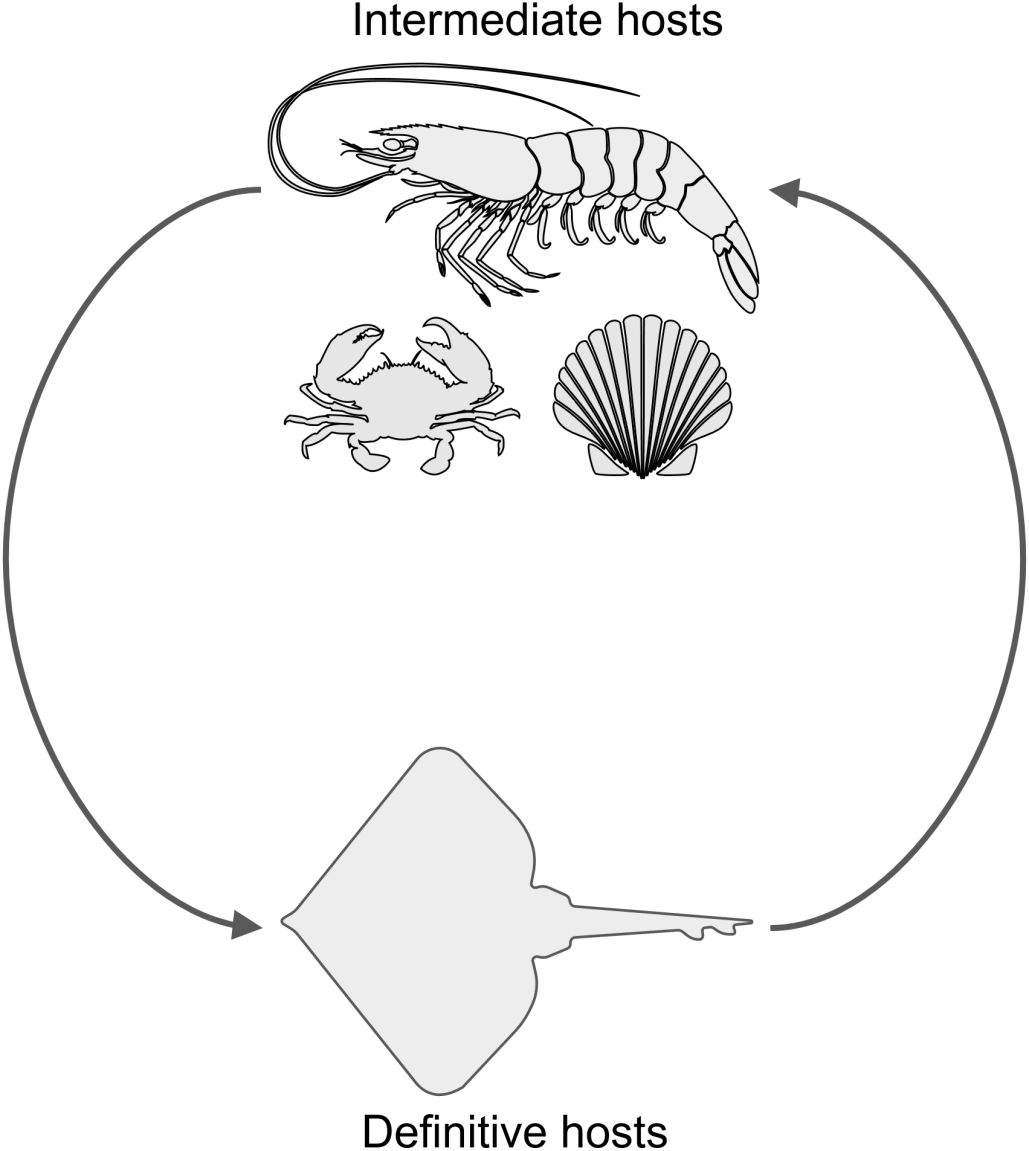
Hypothesized life cycle of *Polypocephalus* sp. Larval stages of cestode tapeworms in the genus *Polypocephalus* infect crustaceans and other invertebrates. These intermediate hosts are presumably ingested by the putative definitive hosts, skates and rays, which are expected to excrete *Polypocephalus* eggs. Images from the Noun Project https://thenounproject.com: shrimp by Jeffrey Qua, crab by Mallory Hawes, scallop by B Barrett, and skate by Örn Smári Gíslason, used under CC BY 3.0 license https://creativecommons.org/licenses/by/3.0/us/.

Another parasite that infects decapod crustaceans where these two species live are larval cestode tapeworms in the genus *Polypocephalus*, which infect white shrimp (*Litopenaeus setiferus*) (Carreon, Faulkes & Fredensborg, 2011; Carreon & Faulkes, 2014). Although the life cycle of species in this genus is not completely worked out, it seems likely that it is a two part life cycle (Fig. 2): crustaceans (Villella, Iversen & Sindermann, 1970; Owens, 1985; Shields, 1992; Hudson & Lester, 1994; Brockerhoff & Jones, 1995; Payne, 2010) and other invertebrates (Cake Jr, 1979) for the larval stage, and elasmobranch fishes (e.g., skates and rays) as the definitive hosts for adults (Butler, 1987; Call, 2007; Koch, 2009). There are reasons that could suggest *Polypocephalus* spp. could be either generalists or specialists. On the one hand, *Polypocephalus* spp. larvae infect multiple species from at least two phyla (Cake Jr, 1979; Owens, 1985; Brockerhoff & Jones, 1995), suggesting that species in this genus are generalists. On the other hand, *Polypocephalus* sp. inhabit the nervous system of crustaceans, and appear to manipulate behaviour in *L. setiferus* (Carreon, Faulkes & Fredensborg, 2011), which are factors that suggest species in this genus are specialists.

*Polypocephalus* sp. is also a candidate for studying the manipulation of host behaviour, because the larval stage infects the neural tissue of their decapod crustacean hosts. Being in or near the nervous system would seem to make such manipulation easier for parasites. In white shrimp, increased infection was correlated with increased activity of the host (Carreon, Faulkes & Fredensborg, 2011), which was hypothesized to be a case of parasite-induced trophic transmission. A trophically transmitted parasite in a digging crustacean might be excepted to change the behaviour of its host so it spends more time above sand (Joseph & Faulkes, 2014). *Litopenaeus setiferus* do dig into sand (Eldred et al., 1961; Fuss Jr, 1964; Pinn & Ansell, 1993), and their increased activity with infection would be consistent with the prediction above.

This paper compares the patterns of infection in *L. benedicti* and *E. benedicti* for both nematode and cestode parasites, and tests whether *Polypocephalus* sp. manipulates the behaviour of *L. benedicti* as they do with shrimp (Carreon, Faulkes & Fredensborg, 2011).

## Methods

Sand crabs (*Lepidopa benedicti*) and mole crabs (*Emerita benedicti*) were collected from the beaches of South Padre Island, Texas by turning over sand with a shovel near, and parallel to, the waterline of the shore (Faulkes, 2017; Murph & Faulkes, 2013). Crabs found in the overturned sand or in the water of the trench created were collected. Individuals were sexed by examining pleopod size (longer in females) and the carapace length was measured with digital calipers. Different individuals were used to study infection by nematodes and *Polypocephalus* sp. To examine infection of nematodes, *E. benedicti* were broken using forceps, and nematodes found in the dissecting dish were counted, following the previous study on *L. benedicti* (Joseph & Faulkes, 2014). To examine infection of *Polypocephalus* sp., individuals were anaesthetised by chilling for ∼20 min on crushed ice, dissected in sea water, and the nerve cord was removed. The nerve cord was cut into smaller sections, which were pinned in dishes lined with Sylgard (Dow Corning, Midland, MI, USA). The nerve cords were dehydrated in a progressive ethanol series (70% ethanol for 5 min, 90% ethanol for 5 min, 100% ethanol for 5 min, then 100% ethanol again for 10 min), cleared in methyl salicylate on a depression slide, viewed under a compound microscope (Olympus CX41), and photographed (Olympus C-5050 Zoom digital camera), following a previous study of *L. setiferus* (Carreon & Faulkes, 2014). In some cases, consecutive images at different focal points in the Z axis were compiled into a single image using Helicon Focus v. 6.7.1 Lite (Helicon Soft Ltd., Kharkiv, Ukraine).

Initial observations of 10 individuals of each species indicated that variation in numbers of parasites infecting *L. benedicti* was sufficient to test whether there was a correlation between infection and host behaviour. Because few *E. benedicti* were infected, and there was very little variation in the number of parasites of those that were infected, their behaviour was not examined.

Behavioural tests were similar to those described in Joseph & Faulkes (2014). Individuals were video-recorded digging in a tank 300 mm wide × 150 mm deep × 200 mm high, filled with ∼40 mm of sand from South Padre Island covered by ∼120 mm of seawater. Video was recorded with an iPad 3 using Coach My Video v. 4.4 (http://www.coachmyvideo.mobi). Individuals were released at the top of the tank, and were filmed until the carapace was covered by sand. The total time was calculated by subtracting the submergence times from release time (rounded down to whole seconds). Individuals made three digging trials, each separated by a 5 min rest period when the animal was not disturbed to minimize habituation. The average of the three trials was used for analysis.

The behaviour of crabs fell into three basic categories. An individual could (1) immediately dig into sand (“direct”), (2) stay above sand by tailflipping and rowing its legs (Faulkes & Paul, 1997) before digging (“swim”), or; (3) remain on the top of the sand, immobile, before digging (“sit”). “Swim” and “sit” are not mutually exclusive. An individual could do both in one trial, in either order. For simplicity of analyses, individuals that both swam and “sat” in their three trials were omitted from analyses that examined individuals.

Descriptive statistical analyses and graphs were made in Origin 2017 (OriginLab Corporation). Nonparametric tests were used for most analyses because of nonhomogenous variation in data distribution. Nonparametric statistical analyses were performed in SPSS v. 23 (IBM, Armonk, NY, USA).

## Results

**Figure 3:**
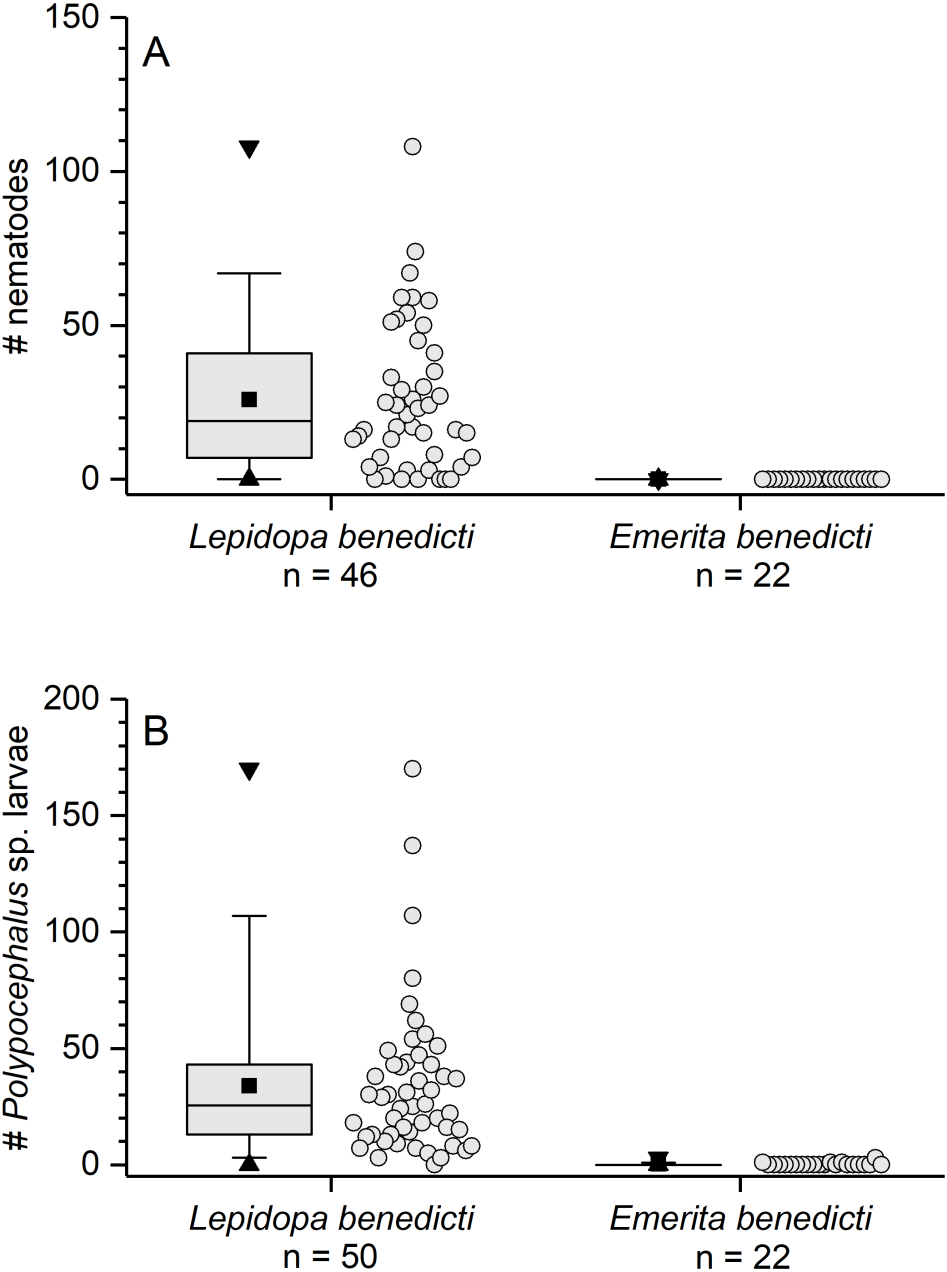
Infection patterns of sand crabs and mole crabs by parasites. (A) Infection of crabs by unidentified nematode species. *Lepidopa benedicti* data redrawn from Joseph & Faulkes (2014). (B) Infection pattern of crabs by *Polypocephalus* sp. larvae. Summary statistics: square, mean; line dividing box, median; box, 50% of data; whiskers, 95% of data; triangles, minimum and maximum. Raw data shown by dots.

**Figure 4:**
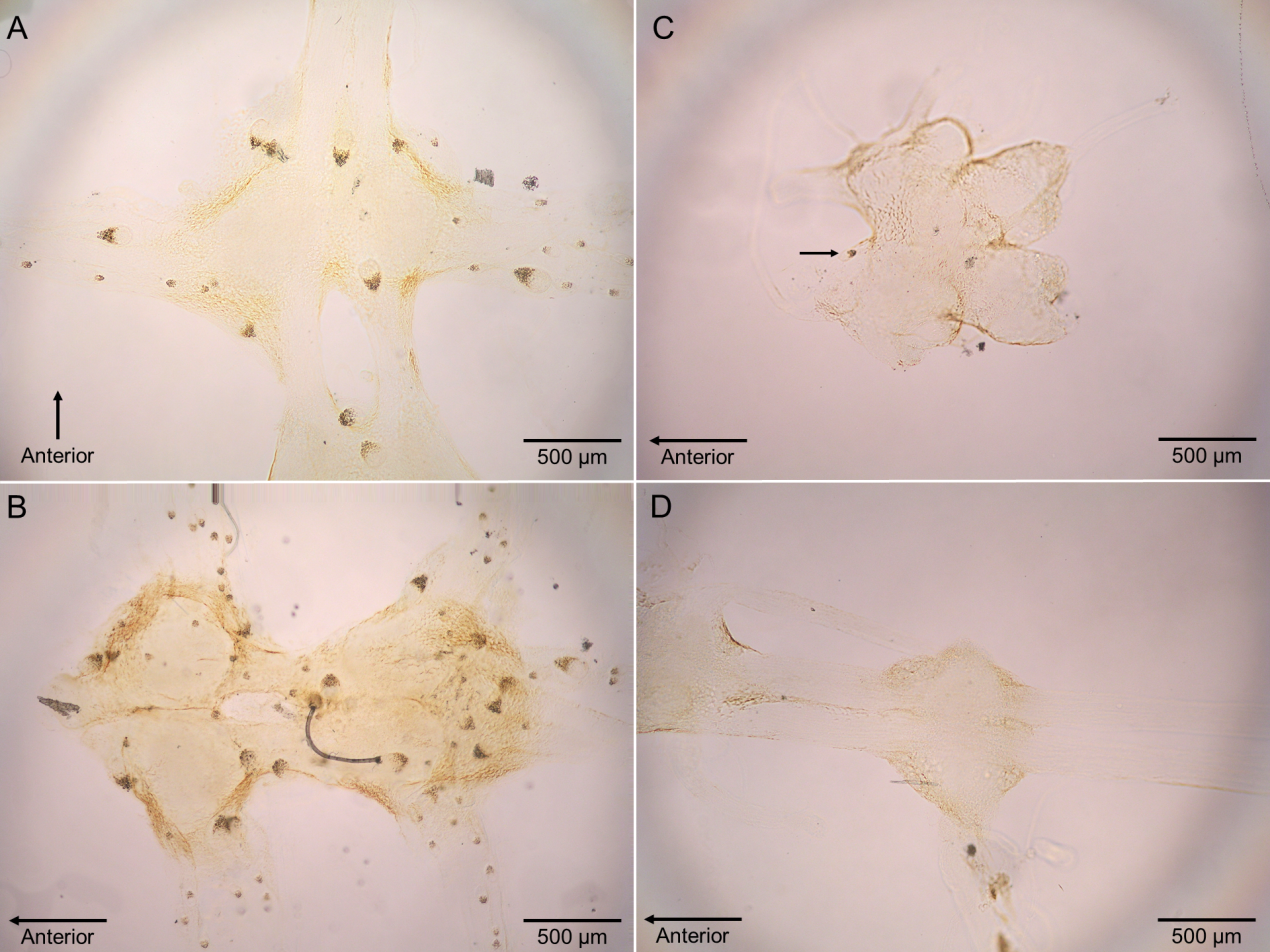
Micrographs of *Polypocephalus* sp. in nervous tissue. (A) *Lepidopa benedicti* thoracic ganglion 2. (B) *Lepidopa benedicti* thoracic ganglia 3, and fused ganglion consisting of thoracic ganglia 4, 5, and abdominal ganglion 1. Different individual than (A). (C) *Emerita benedicti* brain. Arrow indicates single *Polypocephalus* larvae. (D) *Emerita benedicti* thoracic ganglion 1. No *Polypocephalus* sp. larvae in this individual. Different individual than (C). Anterior towards top in (A), and towards left in (B–D).

The previously reported prevalence of nematodes in *L. benedicti* (87.0%, *n* = 46) (Joseph & Faulkes, 2014) was higher than in *E. benedicti* (0.0%, *n* = 22) (Fig. 3A). Similarly, the prevalence of *Polypocephalus* sp. infection in *Lepidopa benedicti* (98.0%, *n* = 50) was higher than in *E. benedicti* (18.2%, *n* = 22) (Fig. 3B). The mean intensity of *Polypocephalus* sp. infection (Figs. 3B and 4) was greater in *L. benedicti* (range = 1–170, mean = 34.5, SD = 33.0, *n* = 49; uninfected animals excluded) than *E. benedicti* (range = 1–3, mean = 1.5, SD = 1.0, *n* = 4; uninfected animals excluded). These differences are not because of the overall size of individuals examined: the average size of *L. benedicti* was smaller than *E. benedicti* (Table 1) in both cases. Because there were so few parasites of either species in *E. benedicti*, all further analyses concern only *L. benedicti*.

*Polypocephalus* sp. larvae were closely associated specifically with neural tissue, including peripheral nerves to appendages (Fig. 4). The larvae often appeared on the surface of ganglia and could sometimes be seen on the dissected nerve cord using a stereomicroscope.

**Figure 5:**
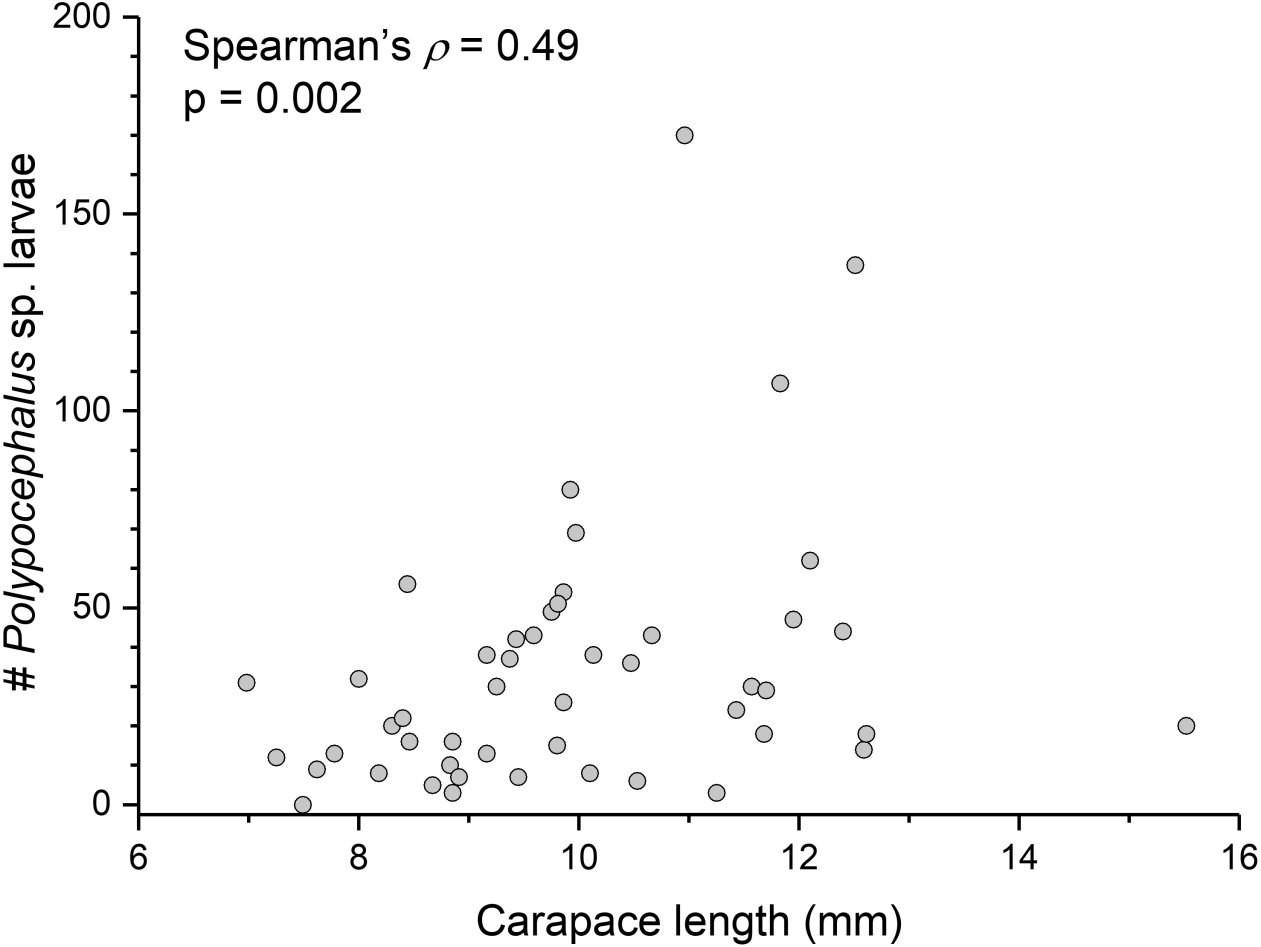
Bigger *Lepidopa benedicti* have more *Polypocephalus* sp. larvae. Relationship between size of *L. benedicti* and intensity of *Polypocephalus* sp. infection.

There is a significant correlation (Spearman’s *ρ* = 0.49, *p* = 0.002, *n* = 38) between *L. benedicti* size and mean intensity of *Polypocephalus* sp. infection (Fig. 5).

**Table 1:** Size of animals used in study.

	*Lepidopa benedicti*			*Emerita benedicti*		
Parasite	Mean carapace length	SD	*n*	Mean carapace length	SD	*n*
Nematode sp.	11.44 mm	2.83	46	13.44 mm	5.45	22
*Polypocephalus* sp.	9.95 mm	1.72	49	18.19 mm	3.65	21

**Figure 6:**
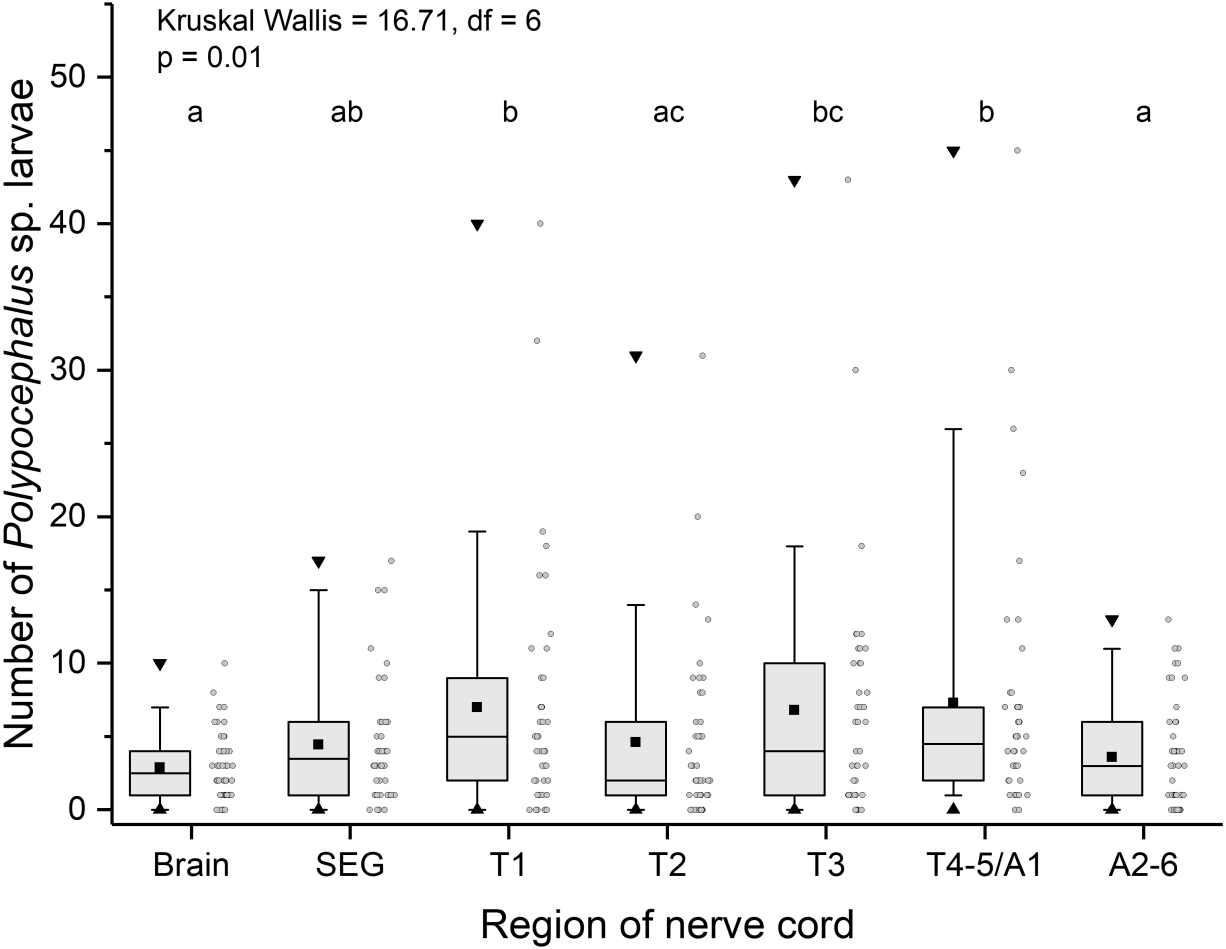
Number of *Polypocephalus* sp. larvae in different regions of the nerve cord in *Lepidopa benedicti*. Summary statistics: square, mean; line dividing box, median; box, 50% of data; whiskers, 95% of data; triangles, minimum and maximum. Raw data shown by dots. Groups sharing a letter do not differ significantly from each other. Sample sizes vary because some ganglia were damaged during dissection. SEG, subesophageal ganglion; T, thoracic ganglion; A, abdominal ganglion.

Like other anomurans, *L. benedicti* have shorter abdomens than familiar decapods like shrimp and crayfish. Because *L. benedicti* are specialized for digging and swimming with thoracic legs 1 through 4, the legs are proportionately more robust. Thoracic leg 5 is very small and used for grooming. These anatomical features are reflected in the relative sizes of the ganglia in *L. benedicti* compared to other decapod crustaceans. The thoracic ganglia associated with thoracic legs 1–4 are substantially larger than abdominal ganglia 2–6. The fourth and fifth thoracic ganglia and the first abdominal ganglion are fused. The number of larvae in the ganglia differed significantly across the nervous system (Kruskal Wallis = 16.71, *df* = 6, *p* = 0.01), with thoracic ganglia containing the most larvae, particularly in highly infected individuals (Fig. 6).

**Figure 7:**
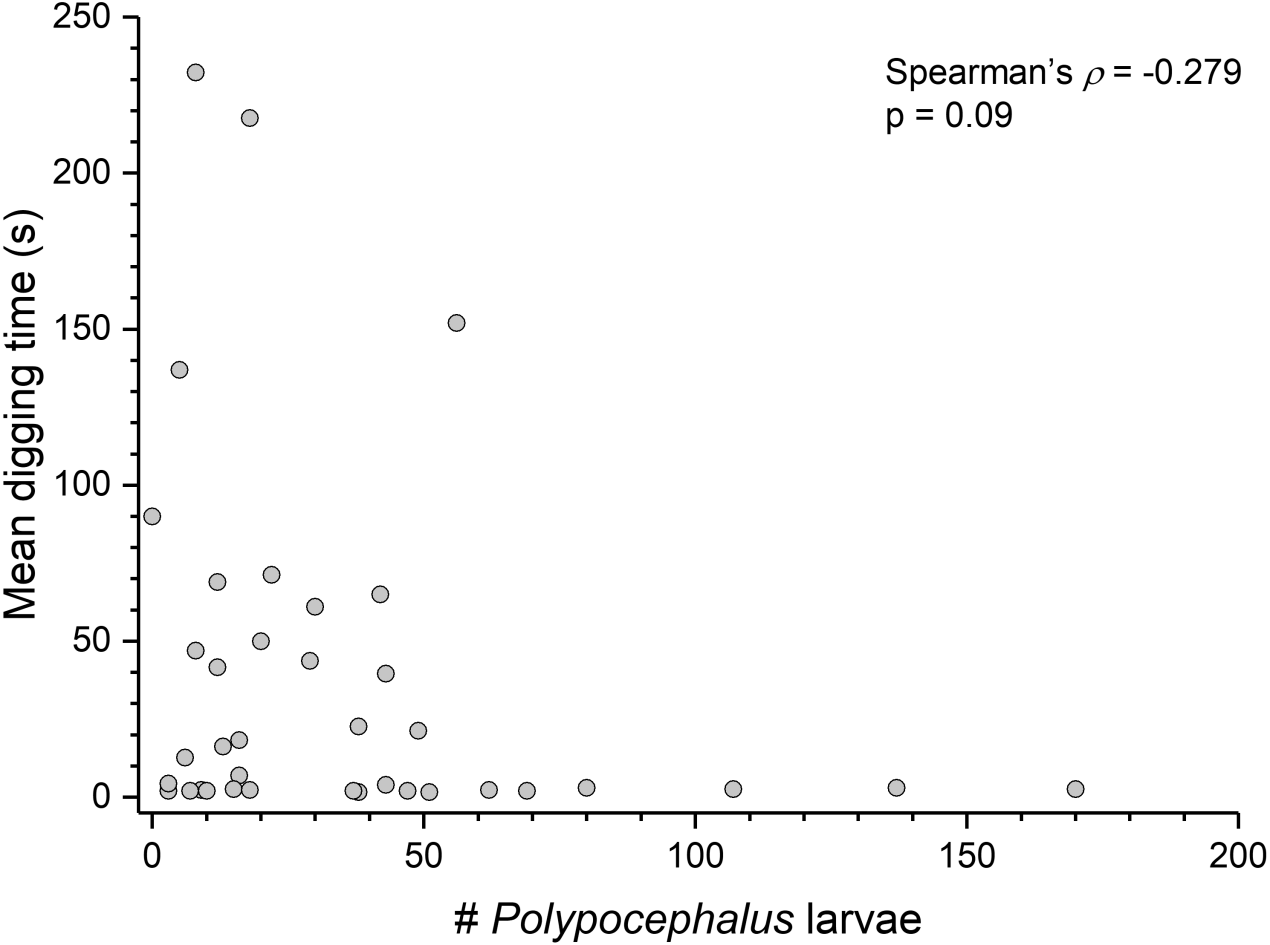
*Polypocephalus* sp. infection does not affect speed of digging in *Lepidopa benedicti*. Relationship between intensity of *Polypocephalus* sp. infection and digging time in *L. benedicti*.

**Figure 8:**
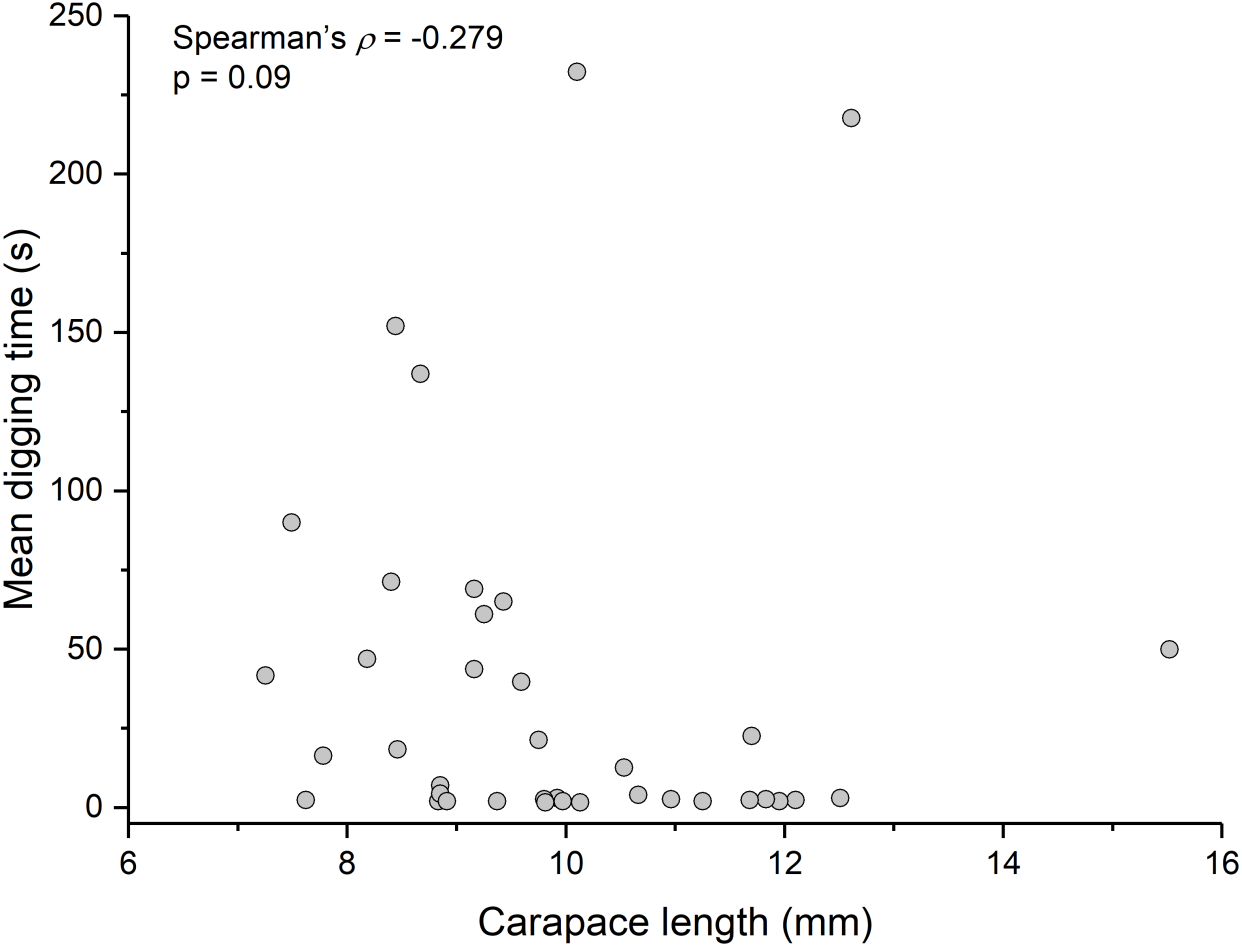
Size does not affect digging time in *Lepidopa benedicti*. Relationship between carapace length and mean digging time in *L. benedicti*.

Contrary to the prediction that more heavily infected animals would spend more time above sand, the mean intensity of *Polypocephalus* sp. infection was not significantly correlated (Spearman’s *ρ* =  − 0.233, *p* = 0.16, *n* = 38) with mean digging time (Fig. 7). Size of *L. benedicti* was not significantly correlated (Spearman’s *ρ* =  − 0.279, *p* = 0.09, *n* = 38) with mean digging time (Fig. 8), confirming previous findings (Joseph & Faulkes, 2014).

**Figure 9:**
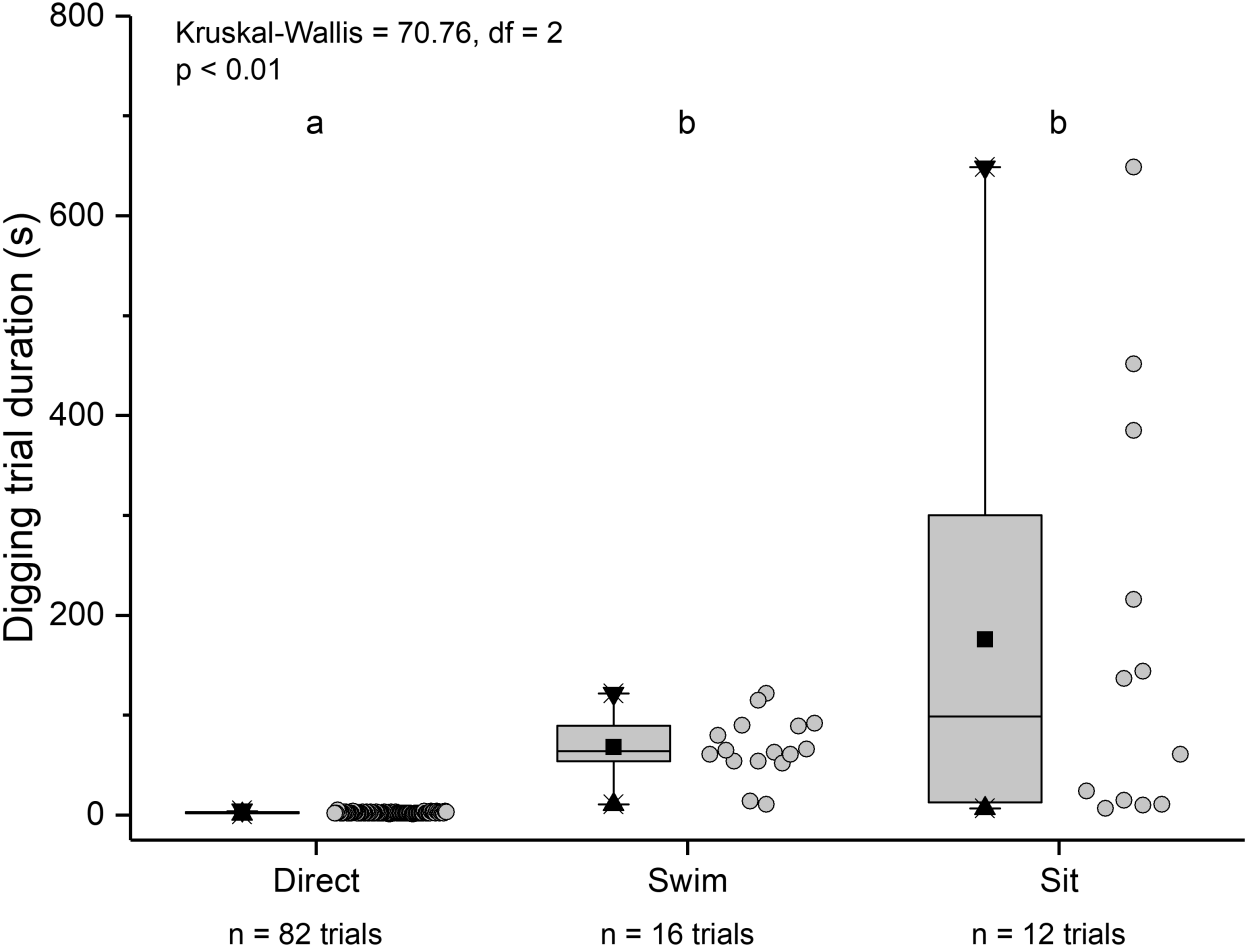
Time above sand for different behaviours by *Lepidopa benedicti*. Duration of individual behaviour trials, grouped by different behaviours. *N* = 110 trials. Four trials involved combinations of sitting and swimming, and are not shown due to their rarity. Summary statistics: square, mean; line dividing box, median; box, 50% of data; whiskers, 95% of data; triangles, minimum and maximum. Raw data shown by dots. Groups sharing a letter do not differ significantly from each other.

The three main behaviours of *L. benedicti* (directly digging into sand, swimming, or remaining stationary, or “sitting”) were significantly different (Kruskal-Wallis = 70.76, *df* = 2, *p* < 0.01) in how long individuals remained above sand (Fig. 9). Swimming above sand and remaining stationary on top of it did not differ significantly in the duration of exposure for sand crabs, although “sitting” times had greater variation, resulted in the longest times that sand crabs were exposed.

**Figure 10:**
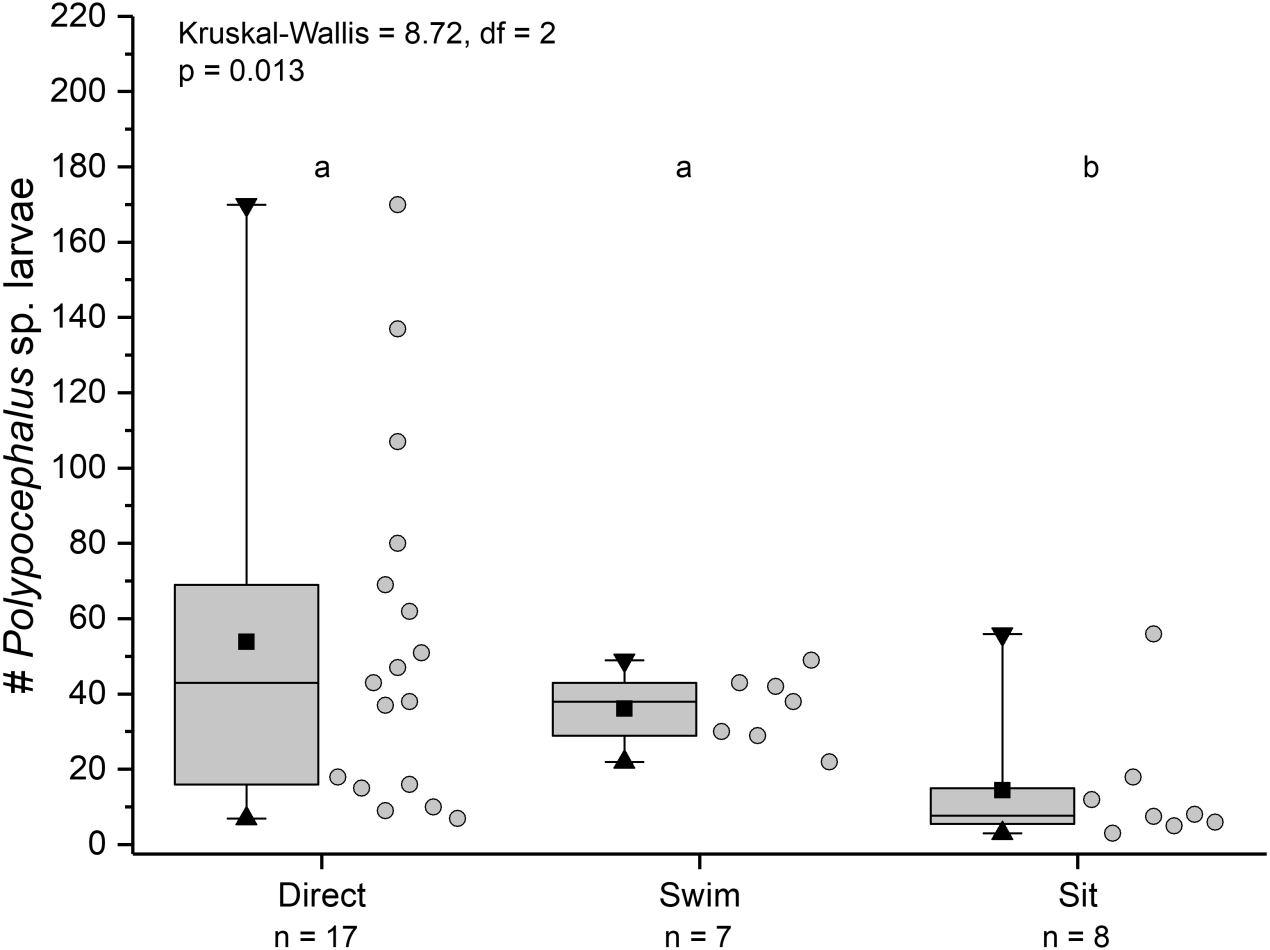
Infection intensity of *Lepidopa benedicti* individuals showing different behaviours. Individuals categorized into three groups: those that always dug directly; those that swam at least once, but never “sat” (i.e., remaining immobile on the surface); those that “sat” at least once, but never swam. Summary statistics: square, mean; line dividing box, median; box, 50% of data; whiskers, 95% of data; triangles, minimum and maximum. Raw data shown by dots. Groups sharing a letter do not differ significantly from each other.

Individuals showing different behaviour patterns had significantly different mean intensities of infection (Kruskal Wallis = 8.72, *df* = 2, *p* = 0.013): animals that “sat” at least once had lower infection intensities than those that swam at least once or always dug directly into sand (Fig. 10).

## Discussion

Two parasite species, an unidentified nematode (Joseph & Faulkes, 2014) and *Polypocephalus* sp., infect *Lepidopa benedicti* with much higher prevalence and intensity than in *Emerita benedicti*. In the case of *Polypocephalus* sp., a high prevalence and intensity also occurs in white shrimp (*L. setiferus*) which also dig into sand (Eldred et al., 1961; Fuss Jr, 1964; Pinn & Ansell, 1993). What distinguishes *E. benedicti* from both *L. benedicti* and *L. setiferus* is the feeding mode. *Emerita* species are filter feeders (Efford, 1966), which *L. benedicti* and *L. setiferus* are not. *Lepidopa* species are probably sediment feeders (Boyko, 2002). This suggests that ingestion is a common route of *Polypocephalus* sp. infection for *L. benedicti* and *L. setiferus*. Presumably, *E. benedicti* avoid infection because they are filtering food from the water column, which is hypothesized to have extremely low numbers of *Polypocephalus* sp. cysts compared to sand and other surfaces.

The lack of parasites in *E. benedicti* in this population is unusual not only because the sympatric *L. benedicti* is infected, but because other populations of *Emerita* species are infected with other parasites (Smith, 2007; Oliva et al., 2008; Kolluru et al., 2011; Violante-Gonzalez et al., 2015; Violante-González et al., 2016). Because this study did not run an entire year, however, it is possible that *E. benedicti* infections vary seasonally or spatially, and that this species is heavily infected at other times or places. That this study did not address whether there any substantial variation in infection rates over time means that the differences in nematode infection in *L. benedicti* and *E. benedicti* should be interpreted with cautiously, because the data for *L. benedicti* (Joseph & Faulkes, 2014) was collected before the data for *E. benedicti* (this study).

In *L. setiferus*, the greatest number of *Polypocephalus* sp. larvae is in the abdominal ganglia (Carreon, Faulkes & Fredensborg, 2011), but in *L. benedicti*, the greatest number is in the thoracic ganglia. This probably reflects which region has the proportionately greater mass of neural tissue available in the two species, although neural mass does not entirely explain distribution patterns across the nervous system (Carreon & Faulkes, 2014). Another difference is that in *L. setiferus*, *Polypocephalus* sp. larvae appeared to be more deeply embedded in neural tissue and were rarely visible under a dissecting microscope until the nerve cord was either squashed or cleared. In *L. benedicti*, larvae were in comparatively superficial positions, and could be seen with dissecting microscopes. There also appeared to be less variation in *Polypocephalus* sp. larval size in *L. setiferus* than *L. benedicti* (compare Fig. 4 here to Fig. 1 in Carreon, Faulkes & Fredensborg, 2011).

*Polypocephalus* sp. does not seem to manipulate *L. benedicti* in a way that would facilitate trophic transmission. Intuitively, one would predict that if *Polypocephalus* sp. were manipulating sand crabs to make them vulnerable to predators, animals with more *Polypocephalus* sp. would be more likely to swim or remain immobile on the top of the sand. In anything, the evidence points towards more heavily infected individuals being more likely to dig into sand immediately. Nevertheless, this result can be viewed as consistent with the results in *L. setiferus*, where higher levels of infection increased activity (Carreon, Faulkes & Fredensborg, 2011). Digging directly into sand and swimming could both be considered higher activity by *L. benedicti*.

The apparent difference in parasite-induced behavioural manipulation in *L. setiferus* and *L. benedicti* has several potential explanations. First, the *Polypocephalus* species infecting *L. setiferus* may not be the same species as the one infecting *L. benedicti*. Although both dig in sand, there are differences in the life history of the two hosts. For example, *L. setiferus* transition from living in seagrass beds (Zimmerman & Minello, 1984) to deeper water as they grow, and change preferences for salinity over their lives (Williams, 1984), whereas *L. benedicti* settle into sand after metamorphosing from a pelagic larva and remain there for their entire lives (Stuck & Truesdale, 1986). These differences in the niches of the host species could be consistent with there being multiple *Polypocephalus* species. Genetic testing will eventually be able to determine if there is one cestode species or multiple species. Second, *L. setiferus* may be the preferred primary host for *Polypocephalus* sp. (perhaps along with other shrimp species), and *L. benedicti* is a non-preferred auxiliary host. The intensity of *Polypocephalus* sp. larvae in *L. setiferus* (mean = 97.7, SD = 102.6; maximum 397; *n* = 53; Carreon, Faulkes & Fredensborg, 2011) is approximately triple that of *L. benedicti* (mean = 34.5, SD = 33.0; maximum 170; *n* = 49; this study). *Litopenaeus setiferus* may be more abundant than *L. benedicti*. *Litopenaeus setiferus* is commercially fished, and annual catches from trawling in the Texas waters of Gulf of Mexico average 7 million pounds per year (Texas Parks and Wildlife, 2002). In contrast, 10 m transects of beach often yield less than 10 *L. benedicti* individuals (Faulkes, 2017; Murph & Faulkes, 2013). *L. benedicti* populations have only been sampled in the swash zone (Faulkes, 2017; Faulkes, 2014) and its abundance in deeper waters is unknown (it has been recorded up to 60 m depth; Boyko, 2002). Nevertheless, it seems plausible that the biomass for *L. benedicti*, and thus its potential as host for *Polypocephalus* sp., is lower than *L. setiferus*. Thus, there may be greater selection pressure for *Polypocephalus* sp. to manipulate its primary host but not secondary ones.

## Additional Information and Declarations

### Data Availability

The following information was supplied regarding data availability: Faulkes, Zen (2017): *Polypocephalus* infection of hippoid crabs. figshare. https://doi.org/10.6084/m9.figshare.5183515.v1.
